# High efficiency Cu_2_MnSnS_4_ thin film solar cells with SnS BSF and CdS ETL layers: A numerical simulation

**DOI:** 10.1016/j.heliyon.2023.e15716

**Published:** 2023-04-25

**Authors:** Ahmmad Isha, Abu Kowsar, Abdul Kuddus, M. Khalid Hossain, Md Hasan Ali, Md Dulal Haque, Md Ferdous Rahman

**Affiliations:** a Institute of Fuel Research and Development (IFRD), Bangladesh Council of Scientific and Industrial Research (BCSIR), Dhaka 1205, Bangladesh; b Department of Electrical and Electronic Engineering, Begum Rokeya University Rangpur, Rangpur 5404, Bangladesh; c Solar Energy Laboratory, University of Rajshahi, Rajshahi 6205, Bangladesh; d Atomic Energy Research Establishment, Bangladesh Atomic Energy Commission, Dhaka 1349, Bangladesh; e Department of Electronics and Communication Engineering, Hajee Mohammad Danesh Science and Technology University, Dinajpur 5200, Bangladesh

**Keywords:** Solar cell, Simulation, Cu_2_MnSnS_4_, SnS, SCAPS-1D

## Abstract

The quaternary compound copper manganese tin sulfide Cu_2_MnSnS_4_ is a potential absorber semiconductor material for fabricating thin film solar cells (TFSC) thanks to their promising optoelectronic parameters. This article numerically investigated the performance of Cu_2_MnSnS_4_ (CMTS)-based TFSC without and with tin sulphide (SnS) back surface field (BSF) thin-film layer. First, the impact of several major influential parameters such as the active material's thickness, doping concentration of photoactive materials, density of bulk and interface defect, working temperature, and metal contact, were studied systematically without a BSF layer. Thereafter, the photovoltaic performance of the optimized pristine cell was further investigated with an SnS as BSF inserted between the absorber (CMTS) with a Platinum back metal of an optimized heterostructure of Cu/ZnO:Al/i-ZnO/n-CdS/p-Cu_2_MnSnS_4_/Pt. Thus, the photoconversion efficiency (*PCE*) of 25.43% with a *J*_SC_ of 34.41nullmA/cm^2^ and *V*_OC_ of 0.883 V was achieved under AM1.5G solar spectrum without SnS BSF layer. Furthermore, an improved *PCE* of 31.4% with a *J*_SC_ of 36.21nullmA/cm^2^ and *V*_OC_ of 1.07 V was achieved with a quantum efficiency of over 85% in the wavelengths of 450–1000 nm by the addition of SnS BSF layer. Thus, this obtained systematic and consistent outcomes reveal immense potential of CMTS with SnS as absorber and BSF, respectively and provide imperious guidance for fabricating highly a massive potential efficient solar cell.

## 1 Introduction

Energy is the driving forces for advancing modern civilization [1]. The demand for this crucial factor is increasing faster to faster and reaching the terawatt level globally by 2050 (389nullTWnullh in the first half of 2022), thereby renewable and green sources of energy are considered as an alternative with worldwide efforts to mitigate such huge energy demand ensuring greenery climate [2,3]. Among the energy sources, solar energy is a rapidly installed green energy endeavor for generating renewable electric power directly. The crystalline Si (c-Si) modules have occupied a market share of 95% of the mainstream global PV market till the early 21st century [4,5]. However, Si-based solar photovoltaic cells possessing a thicker absorber film of ∼100 μm with several accompanying photoactive layers limit their further technological advancement and applications [6,7]. On the other side, thin film PV technology uses components of a few layers to bulk semiconductors, i.e., several hundred nm to a few μm thicknesses, including cadmium telluride (CdTe), copper-based zinc tin sulfide compound (CZTS) and indium gallium selenide compound (CIGS), molybdenum disulfide (MoS_2_), tungsten disulfide (WS_2_), silicide, and perovskite-based solar cells (SC) [[6], [7], [8], [9]]. The efficiency *η* of ∼23.35, ∼11.0, ∼12.6, and ∼22.0% for CIGS, CZTS, CZTSSe, and CdTe thin films reported under the global air mass AM1.5G solar light irradiation [10]. Further, the highest mentioned *PCE* of ∼24.2% for perovskite solar cells was achieved recently, regardless of its instability [[11], [12], [13]]. Although, the CdTe-based SC showed potential for industrial-scale production, having a photovoltaic market share of almost 20 GW higher than the CIGS and a-SiH-based photovoltaic solar cell [11]. However, both elements Indium (In) and Gallium (Ga) in absorber of CIGS are scarce elements of earth, and Cadmium (Cd) is a critical carcinogenic [12,14,15], potentially hindering further large-scale development. However, a promising member of the quaternary compound semiconductor group, Cu_2_MnSnS_4_ is a cost-effective, environmentally benign, earth-favored compound, which possesses a tunable direct bandgap in the range from 1.49 to 1.51 eV and high coefficient of absorption in visible region (α ≥ 10^4^ cm^−1^) [13,[16], [17], [18], [19]]. The computational efficiency of 16.5–20.26% obtained in an i-ZnO/CdS/Cu_2_MnSnS_4_ and ZnO:Al/i-ZnO/buffer (ZnO, ZnS, CdS)/CMTS heterostructure recently [6,20]. However, a detailed investigation of the CMTS-absorber layer with a favorable BSF layer, buffer layer, satisfactory metal contact, and consistency has been unexplored.

In this present scientific report, a detailed numerical study on the impact of the layer's parameter of used photoactive materials with Cu/ZnO: Al/i-ZnO/CdS/CMTS/Pt heterostructure was performed without SnS BSF layer and followed by the photovoltaic performance of the optimized cell was performed at varying major parameters; thickness, doping density, interface and defect level, DOS function and back contact. Furthermore, a comparative study of the proposed cells and previous reports was studied afterward. This detailed study revealed that a CMTS absorber with SnS BSF and CdS window layer has a strong potential and provided systematic instruction for the experimental demonstration of high-performance, low-cost CMTS-based TFSCs.

## 2 Methodology, device structure, and material parameters

The simulation approach is essential to immediately understand the physical properties and operation of the photovoltaic devices and the behaviour of each device parameter without spending huge currency and time. This study investigated CMTS-based TFSCs with (Cu/ZnO: Al/i-ZnO/CdS/CMTS/Pt) heterostructure using Solar Cell Capacitance Simulator (SCAPS-1D) tool. This computer-aided software tool can solve semiconductor equations of optoelectronic devices. It determines quasi-Fermi levels and electrostatic potential of *e* and *h*, and thereby it calculates other parameters; carrier concentration, electric field, and recombination level etc. [21]. Furthermore, it allows the modelling of varieties of electronic structures, including homojunction, multijunction, heterojunction, and even Schottky barriers designed using specified and practically obtained input parameters (electrical and optical) of the simulation [22]. Currently, there is a list of simulation software, such as PC1D, ATLAS, Sentaurus TCAD, wxAMPS, AFORS-HET, AMPS-1D, and SCPAS-1D, have been utilized to design and evaluate overall PV performance [3,[23], [24], [25], [26]]. Among them, SCPAS-1D showed a strong potential for modelling and simulation of varieties structures of solar cell devices with versatile parameters; capacitance-voltage, capacitance-frequency, series and shunt resistance, recombination mechanism, working temperature, metal contacts, as well as both single and batch calculations by a simple and user-friendly interface and fast speed. SCAPS-1D has several limitations, including only one-dimensional operation. However, there are several challenges in the SCAPS-1D simulator, such as limited to seven layers, unstable performance for a secondary barrier or *n-p* (instead of *p-n*) junction, and divergence error when the simulation step is unlimited [27]. The reference parameters of used photoactive materials used in this simulation study have been abridged in Table 1 [28,29].

**Table 1:** The optoelectronic parameters used for this numerical simulation study and investigation.

Materials/Parameters	ZnO:Al [30,31]	i-ZnO [30,32,33]	*n*-CdS [34,35]	*p*-CMTS [6]	*p*^*+*^-SnS [36]
Thickness, μm	0.20	0.05	0.05	0.8	0.1
Electron affinity, |e (eV)	4.6	4.4	4.4	4.35	4.1
Bandgap, *E*_g_ (eV)	3.3	3.3	2.42	1.20	1.6
Energy level w.r.t reference, (eV)	1.65	1.65	1.2	0.8	0.6
Dielectric constant, κ	7.8	7.8	8.28	7.6	13
Donor concentration, *N*_d_ (cm^−3^)	1 × 10^20^	1 × 10^15^	1 × 10^17^	–	–
Acceptor concentration, *N*_a_ (cm^−3^)	–	–	–	1 × 10^16^	1 × 10^19^
Effective DOS of CB *N*_c_, (cm^−3^)	2.2 × 10^18^	2.2 × 10^18^	1.7 × 10^19^	2.2 × 10^18^	1.18 × 10^18^
Effective DOS of VB, *N*_v_ (cm^−3^)	1.8 × 10^19^	1.8 × 10^19^	2.4 × 10^18^	1.8 × 10^19^	4.46 × 10^18^
Total bulk defect density, *N*_t_ (cm^−3^)	1 × 10^14^	1 × 10^14^	1 × 10^16^	1 × 10^11^	1 × 10^16^
Electron mobility, *μ*_n_ (cm^2^ V^−1^s^−1^)	160	160	350	0.16	15
Hole mobility, *μ*_p_ (cm^2^ V^−1^s^−1^)	40	40	50	0.16	100
Capture cross-section area of e, (cm^2^)	1 × 10^−15^	1 × 10^−12^	1 × 10^−15^	1 × 10^−15^	1 × 10^−16^
Capture cross-section area of h, (cm^2^)	1 × 10^−15^	1 × 10^−15^	1 × 10^−15^	1 × 10^−15^	1 × 10^−16^
Total interfaces defect density, *N*_t_ (cm^−3^)	neutral: 1 × 10^11^				

**Fig. 1:**
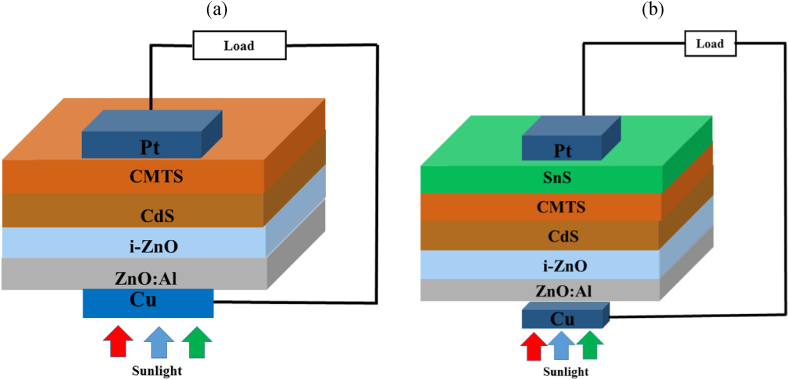
Schematic diagram of CMTS- based solar cells (a) Pristine cell combination; (b) Cell combination incorporating SnS BSF layer.

**Fig. 2:**
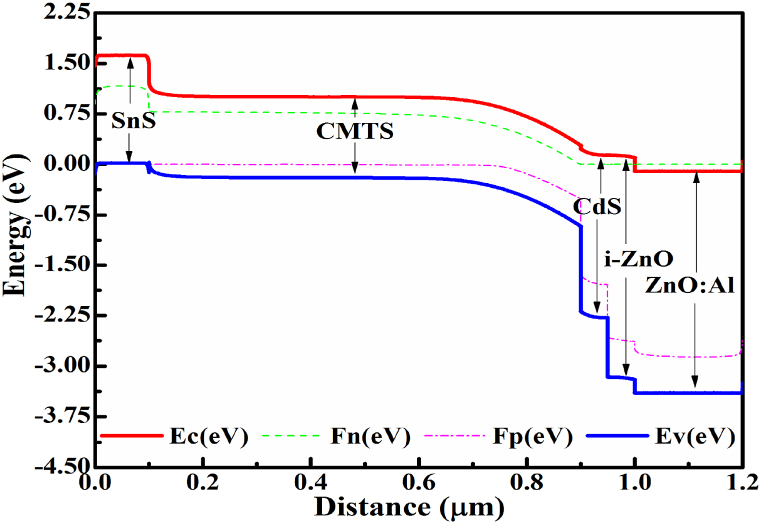
The bandgap diagram representation of the suggested CMTS-based solar cell incorporating the SnS BSF.

Fig. 1 (a) and (b) show a schematic diagram of Cu/ZnO: Al/i-ZnO/CdS/Cu_2_MnSnS_4_/Pt heterojunction without and with SnS as BSF and corresponding band alignment, respectively. In the proposed cell layout, stacked ZnO: Al/i-ZnO was utilized as transparent conductive oxide (TCO), CdS as window/buffer, Cu_2_MnSnS_4_ (CMTS) as absorber and SnS as BSF/hole transport layers. The device performance was determined under airmass AM1.5G solar irradiance at normal sunlight exposure (1000 Wm^−2^) at 300 K operating temperature. Single defects (above E_V_ of 0.6 and 0.1 eV from reference) with uniform distribution were introduced in each semiconductor layer as bulk defects. The corresponding bandgap alignment of this proposed CMTS-based solar cell is depicted in Fig. 2. The recombination at two interfaces of CMTS/CdS and CMTS/SnS is taken into account; therefore, neutral interface defects have also been utilized. Further, from previous literature, the R_sh_ and R_s_ resistances were kept constant at 10^5^ Ωcm^2^ & ∼0.1 Ωcm^2^, respectively [37,38]. The electrical and optical data, including the absorption coefficient, were collected from reported literature and experimental outcomes [[30], [39], [40], [41], [42], [43]].

## 3 Results and discussion

The effect of the major influential parameters of photoactive material layers, such as layer thickness, dopant concentration, and interface defect density, has been explored systematically in this work. The following sub-sections demonstrate the impacts of each parameter on CMTS-absorbed-based solar cells sequentially.

### 3.1 Impact of the thickness and acceptor concentration of CMTS absorber

**Fig. 3:**
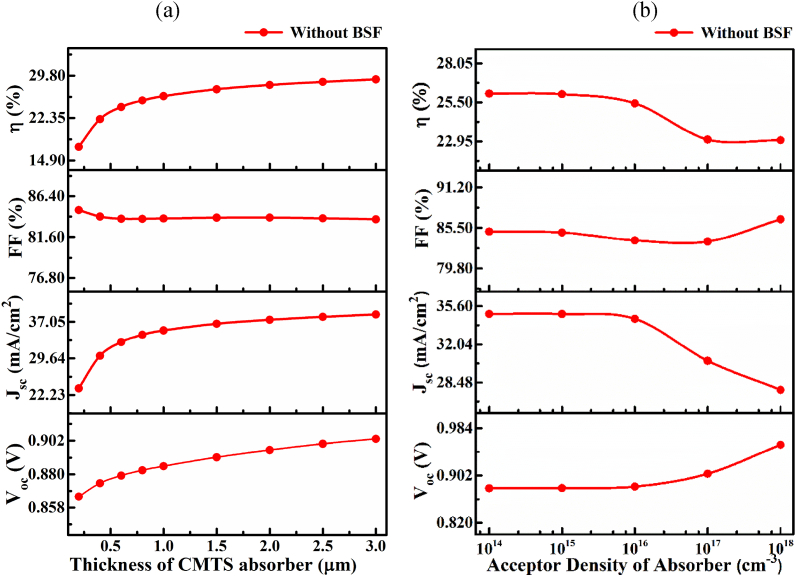
Solar cell performance at a different CMTS (a) absorber thickness, and (b) acceptor density.

Among several parameters, the layer thickness and acceptor carrier density are the most vital parameters on solar cell. Fig. 3(a) shows the impact of the CMTS absorber layer thickness in a range of 0.1–3.0nullμmnullat a fixed acceptor concentration *N*_A_ of 10^16^ cm^−3^. The cell efficiency of CMTS solar cell increased systematically with the increase of layer thickness. The photocurrent *J*_SC_ increased from 23.566 to 38.559nullmA/cm^2^ exponentially with increases of CMTS layer thickness from ∼0.1 to 1.0 μm, and it reaches an almost saturated value for further increase of layer thickness beyond 1.5 μm. Consequently, the photo-conversion efficiency increased from 17.28 to 29.15% when the layer thickness of ∼1.0 μm. Although the higher absorption of an incident photon at a thicker (>1.0 μm) absorber layer is obtained, recombination due to longer diffusion length compared with carrier lifetime may be dominant. Thus, the layer thickness of 0.8 μm was selected as an optimum for further study of cell performance without and with the SnS BSF layer material.

The impact of the *N*_A_ of the CMTS absorber layer ranging from 1 × 10^14^ to 1 × 10^18^ cm^−3^nullat a fixed thickness of 0.8 μm is demonstrated in Fig. 3(b**)**. The *V*_OC_ increased from 0.879 to 0.955 V sub-linearly when the doping density *N*_A_ increased from 1 × 10^14^–1 × 10^18^ cm^−3^. An increase in acceptor concentration enhanced the build-in potential forming a strong junction at the CdS and CMTS interface; consequently, the *V*_OC_ improved significantly. The photocurrent *J*_SC_ is almost unchanged up to a *N*_A_ of 1 × 10^16^ cm^−3^ and, thereafter, decreased linearly from 34.864 to 27.79nullmA/cm^2^ at a N_A_ decrease from 1 × 10^16^–1 × 10^18^ cm^−3^. This decrease in photocurrent at higher acceptor concentrations is due to increased Auger recombination at higher carrier concentrations. Further, the *FF* of ∼84% changes insignificantly up to the acceptor concentration of 1 × 10^15^ cm^−3^, but it is decreased slightly for further increase in *N*_A_. A similar consequence of acceptor concentration was also observed in previous reports [15,24]. Thus, the acceptor concentration *N*_A_ of 1 × 10^16^ cm^−3^ and the absorber layer thickness of 0.8 μm obtained an optimum value, which was used for further investigation.

### 3.2 Impact of CdS layer thickness and donor density

**Fig. 4:**
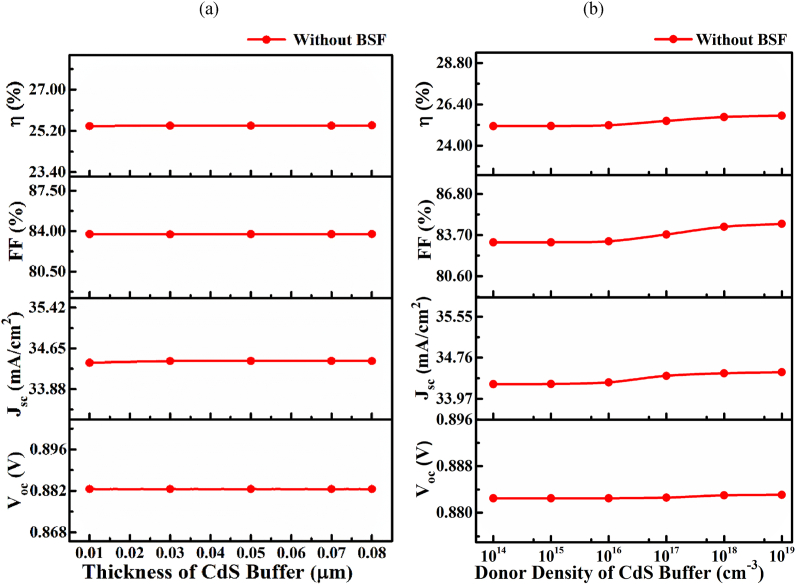
(a) Solar cell performance at different CdS layers thickness, (b) different donor density without SnS BSF.

The buffer layer of a TFSC is crucial for separating electrons and holes at either side of the device. Compared to the absorber material, a higher band gap buffer material conventionally used for passing the incident light to the junction area results in an improved electron-hole pair generation by higher photon absorption. In addition, a selective carrier (electron) flow from the photoactive region of the cell to the outer metal electrode (front contact) also offered an efficient collection of photogenerated carriers. A thinner as less as possible thickness is required to be to allow maximum incident light freely. Nevertheless, a very thinner thickness (>50 nm) may cause a noticeable leakage current [44]. Therefore, this study's buffer layer thickness changed from 0.01 m to 0.08 μm [36]. Fig. 4(a) exhibits the impact of CdS buffer thickness on solar cell performance parameters corresponding to a layer thickness of 0.01–0.08 μm. The PV parameters of *V*_OC_, *J*_SC_, and *FF* change slightly; therefore, the PCE is almost unchanged with increasing CdS layer thickness. Thinner thickness compared with total active layer (∼1.2 μm) CdS layer having wide band gap and higher donor concentration N_D_ of 1 × 10^17^ cm^−3^. Considering the trade-off between cell performance and film growth challenges, the buffer layer thickness was adjusted at 0.05 μm. Fig. 4(b) shows the impact of CdS donor concentration on cell performance for the donor concentration *N*_D_ of 1 × 10^14^–1 × 10^19^ cm^−3^nullat a fixed layer thickness of 0.05 μm. The *V*_OC_ and *J*_*SC*_ are found to be almost unchanged, while the *FF* is increased slightly from 83.15 to 84.54% during donor density increase from 1 × 10^16^–1 × 10^19^ cm^−3^, as a similar trend is observed in layer thickness-dependent response.

This slight improvement in FF refers to an enhancement of build-in potential at a higher concentration *N*_D_ of 1 × 10^16^–1 × 10^19^ cm^−3^. Thus, a wide band gap CdS layer shows the highest *PCE* of 25.43% at an optimized thickness of 0.05 μm.

In addition, the *PCE* was found in the range of 25.60–25.28% with *J*_SC_ of 34.63–34.21nullmA/cm^2^, *V*_OC_ of 0.883–0.8824 V, and *FF* of 83.74–83.73% for varying ZnO: Al layer thickness in the rage of 0.1–0.4 μm. Further, the PCE is in the range of 25.22–25.43% with *J*_SC_ of 34.13–34.41nullmA/cm^2^, *V*_*OC*_ of 0.8824–0.8826 V, and *FF* of 83.72–83.74% for the bandgap *E*_g_ of 3.0–3.5 eV was obtained while the *PCE* in the range of 23.04–25.43% with *J*_SC_ of 34.32–34.41nullmA/cm^2^, *V*_OC_ of 0.8743–0.8826 V and *FF* of 76.68–83.74% for the varied electron affinity of 4.1–4.6 eV. Considering this insignificant change in PV performance, an optimized ZnO:Al with a layer thickness of 0.2 μm and a carrier concentration of 1 × 10^20^ cm^−3^ was selected as a TCO material for the simulation throughout this study.

### 3.3 Impact of bulk defect density of CMTS absorber, and CdS buffer layer BSF

**Fig. 5:**
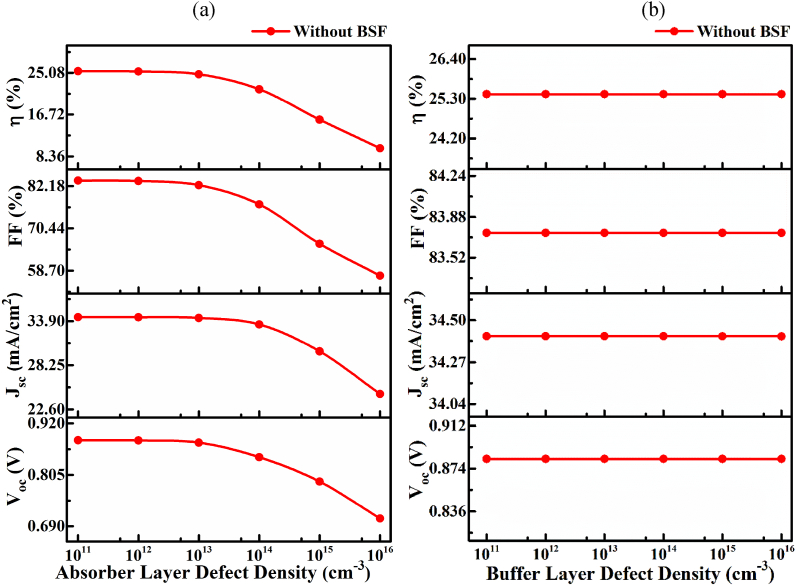
Photovoltaic performance (PV) performance at different bulk defect densities of (a) CMTS absorber and (b) CdS buffer layers.

Fig. 5(a) shows the impact of the bulk neutral defect associated with CMTS absorber material, which includes vacancies, antisites, and interstitials defects, on cell performance from 1 × 10^11^ to 1 × 10^16^ cm^−3^. The *V*_OC_ decreased from 0.8826 to 0.7076 V, *J*_SC_ from 34.411 to 24.579nullmA/cm^2^, *FF* from 83.74 to 57.3%, and consequently, the efficiency decreased from 25.43 to 9.97% with Cu/ZnO:Al/i-ZnO/CdS/Cu_2_MnSnS_4_/Pt solar cell when defect density *N*_t_ increasing from 1 × 10^11^ to 1 × 10^16^ cm^−3^. The occupancy of defects in the forbidden energy gap acts as Shockley-Read-Hall (SRH) recombination sites that deteriorate the cell performance noticeably. The primary origin of the defect formation between two active adjacent material layers appears due to be the lattice mismatch between them [37,38]. Therefore, the CMTS absorber's bulk defect density ≤1 × 10^11^ cm^−3^ showed the highest solar cell performance. Fig. 5(b) depicts the impact of the bulk neutral defect associated with CdS buffer material on solar cell performance for defect density from 1 × 10^11^ to 1 × 10^16^ cm^−3^. The *V*_OC_, *J*_SC_, and *FF* have no significant change in defect density up to 1 × 10^16^ cm^−3^. The defect density of CdS buffer affects negligibly owing to thinner thickness with a high bandgap and concentration, as observed in a previous report []. However, the highest efficiency of ∼25.4 % with open circuit voltage (V_OC_) of 0.88 V, short-circuit current (J_SC_) of 34.4nullmA/cm^2^, and fill-factor (FF) of ∼83.7 % was found from optimized reference solar cell with Cu/ZnO: Al/i-ZnO/CdS/Cu_2_MnSnS_4_/Pt configuration at the thickness of 0.8 μm and 0.05 μm and carrier density of 1 × 10^16^ and 1 × 10^17^ cm^−3^ with a defect density of 1 × 10^11^ cm^−3^ and 1 × 10^16^ cm^−3^ for CMTS absorber and CdS window layer, respectively. A systematic investigation has been performed to improve the optimized cell configuration further using the SnS BSF layer in the latter part.

### 3.4 Impact of SnS BSF layer thickness and acceptor density

**Fig. 6:**
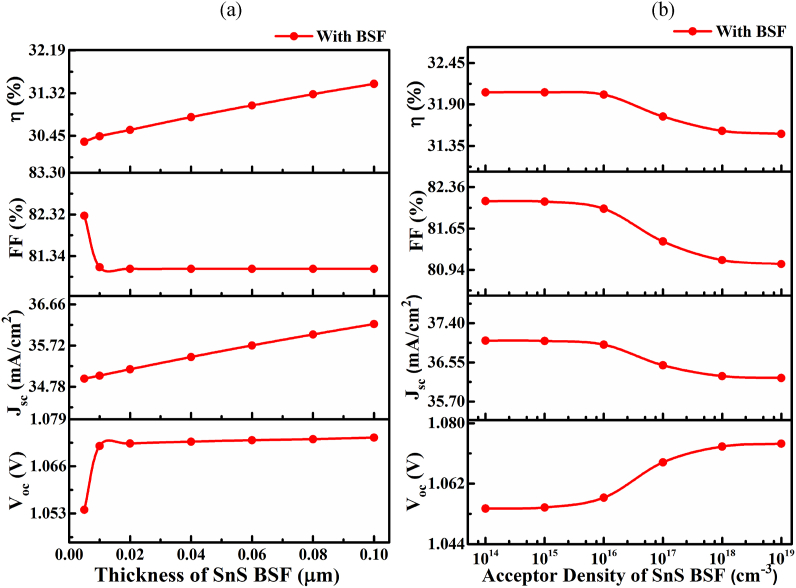
The impact of (a) the thickness and (b) acceptor density of SnS BSF layer on CMTS TFSC.

The accumulation of light-affected charge carriers in the absorber film depends heavily on the buffer and BSF. The light-generated excitons developed in the absorber reached the interfaces and were separated by build-in-field. The electrons are conveyed to the ETL and holes to the BSF layers; in contrast, the ETL blocks the holes while BSF blocks the electrons simultaneously. Therefore, the doping level of *N*_A_ and thickness of the BSF play a vital role in effectively transporting selective carriers (holes) as ETL for electrons. The effect of the layer thickness of SnS BSF layer on proposed solar cell performance having heterostructure with SnS BSF at a fixed *N*_A_ of 1 × 10^19^ cm^−3^ is demonstrated in Fig. 6(a). The *J*_SC_ and *V*_OC_ increased markedly from 34.964 to 36.21nullmA/cm^2^ and 1.054–1.073 V, respectively, while the *FF* decreased slightly from 82.29 to 81.04%, resulting in the efficiency increases from 30.33 to 31.51% with increasing the BSF layer thickness ranging from 0.05 to 0.1 μm. An increment in *J*_SC_ and *V*_OC_ with increasing BSF layer thickness revealed a decrease rate of recombination of photo-generated charge carriers in the active area of the solar cell .

Fig. 6(b) demonstrates the effect of the BSF carrier density on solar cell performance. Therefore, the cell *PCE* is retained approximately constant up to the *N*_A_ of ≤1 × 10^16^ cm^−3^. Further, the *V*_OC_ increased from 1.054 to 1.073 V, while the *J*_SC_, *FF*, and *PCE* decreased from 37.021 to 36.21nullmA/cm^2^, 82.12 to 81.04%, and 32.06 to 31.51%, respectively, with increasing the BSF carrier concertation from 1 × 10^16^ to 1 × 10^19^ cm^−3^nullat a fixed thickness of 0.1 μm. The electrical field at the BSF/CMTS interface created by high acceptor concentration blocks the surge of minority electrons lowering the interface recombination. Furthermore, the lower contact resistance with insignificant Schottky barrier at the SnS/Pt interface owing to the metal-like behavior of the highly doped (*N*_A_ of 1 × 10^19^ cm^−3^) SnS layer enhances the effective hole transportation. Thus, the maximum efficiency *η* of 31.51% (24% higher than the reference solar cell ) and *J*_SC_ of 36.21nullmA/cm^2,^ *V*_OC_ of 1.073 V, and *FF* of 81.04% were obtained by inserting the SnS BSF layer at an adjusted SnS layer thickness of 0.1 μm and carrier density *N*_A_ of 1 × 10^19^ cm^−3^.

### 3.5 Impact of the interfaces defect density

**Fig. 7:**
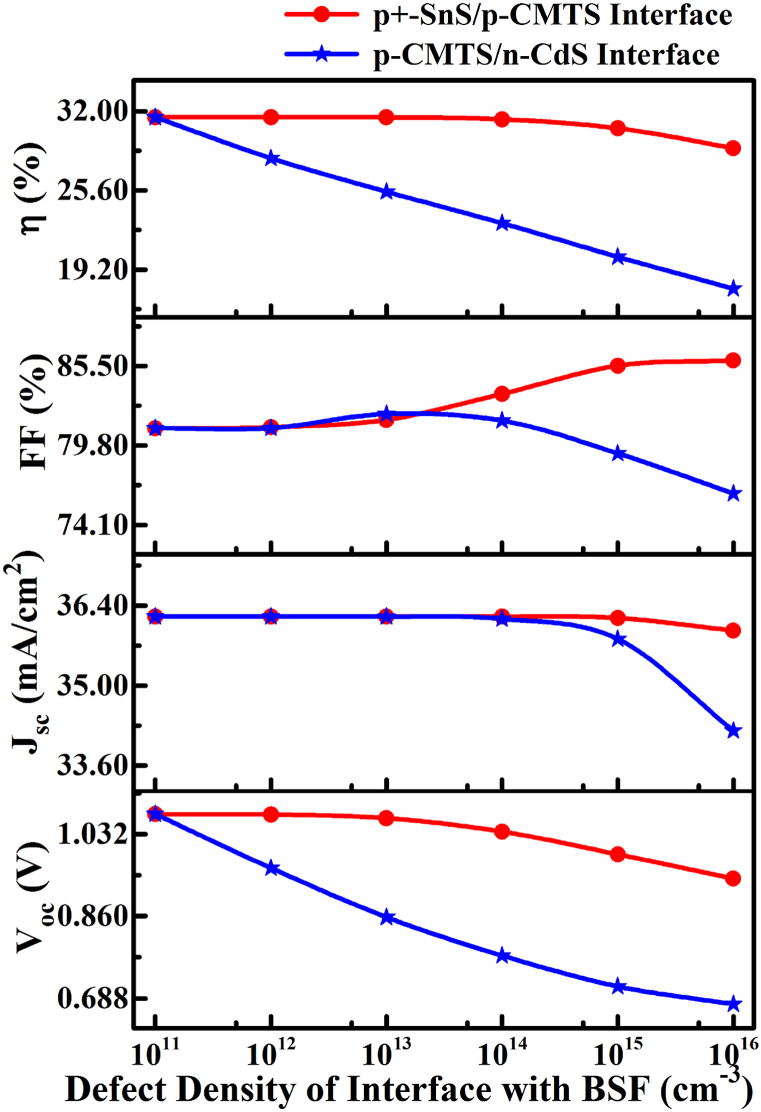
Impact of the defect density of *p*-CMTS/*n*-CdS and *p*^*+*^-SnS/*p*-CMTS interfaces on PV performance with SnS BSF layer.

Interfaces formed between different semiconductor layers are used as active materials with a network of dislocations between the absorber semiconductor and buffer layers. Moreover, the inter-circulation of the particle of the elements within adjacent layer materials through the device growth procedure leads to structural flaws in the interfaces of solar cell [40,41]. Herein, the Cu/ZnO: Al/i-ZnO/CdS/Cu_2_MnSnS_4_/SnS/Pt proposed solar cell performance is potentially suffered by interfacial defects in a hetero-structure configuration. Fig. 7 shows the simulation results of solar cell properties as a function of interface defect density of *p*-CMTS/*n*-CdS and *p*^*+*^-SnS/*p*-CMTS interfaces in the range of 10^11^–10^16^ cm^−3^. The cell performance markedly decreased with the increase in defect density of the *p*-CMTS/*n*-CdS interface. The *V*_OC_ decreased from 1.073 to 0.676 V, *J*_SC_ from 36.21 to 34.21nullmA/cm^2^, and the *FF* from 81.04 to 76.36%, leading to a decrease of the *PCE* from 31.51 to 17.66% when defect density increased from 1 × 10^11^–1 × 10^16^ cm^−3^. On the other hand, the *V*_OC_ decreased from 1.073 to 0.938 V; consequently, the η decreased from 31.51 to 29% with no mentionable differences in *J*_SC_ for *p*^*+*^-SnS/*p*-CMTS interfaces. However, a decreasing rate of cell parameters for *p*^*+*^-SnS/*p*-CMTS interfaces is lower than *p*-CMTS/*n-*CdS may occur owing to the higher carrier concentration in the *p*^*+*^-SnS (metal-like) BSF layer. Simulation results revealed that the *PCE* of >31 % can be obtained at a defect density *N*_t_ of ≤10^11^ cm^−3^. So far, the change in FF with defect density may be demonstrated by a collective change in *V*_OC_ and *J*_SC_ compared to changes in *V*_MPP_ and *J*_MPP_, as given in equation (1) [45].

(1) FF= (V_MPP_ × J_MPP_) / (V_OC_ × J_SC_)

### 3.6 Optimized solar cell

**Fig. 8:**
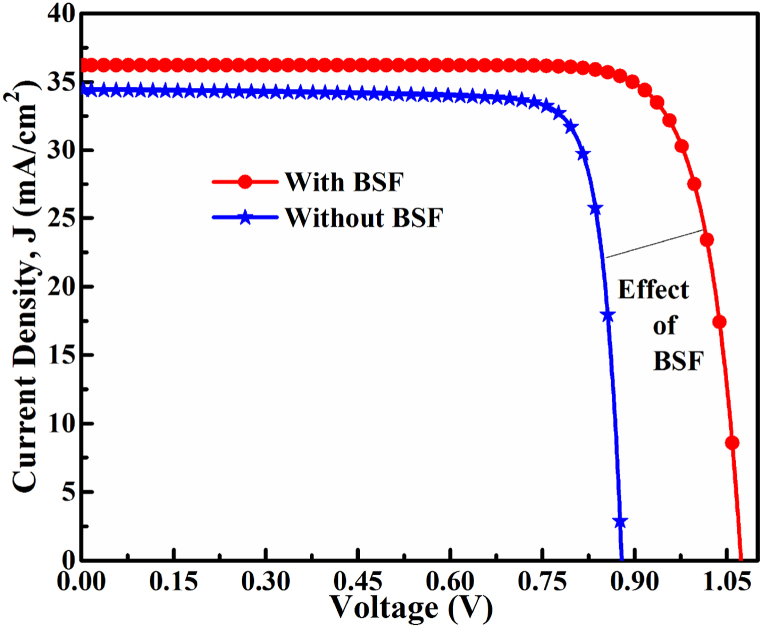
*J-V* characteristics of Cu/ZnO:Al/i-ZnO/*n*-CdS/*p*-CMTS/Pt solar cells without and with SnS BSF.

Fig. 8 shows an optimized *J-V* characteristic curve of the CMTS absorber-based proposed solar cell partnered with CdS as a buffer layer. In *J-V* characteristics, the PCE of 25.43% with the photocurrent (*J*_SC_) of 34.412nullmA/cm^2^, *V*_OC_ 0.882 with *FF* of ∼84 % was found in optimized reference heterostructure (without SnS), while the *PCE* of 31.51% with the photocurrent (*J*_SC_) 36.21nullmA/cm^2^, *V*_OC_ 1.0739 V, and the *FF* of ∼81 % after sandwiching *p*^*+*^-SnS. Optimized results revealed the thickness of 0.05, 0.8, and 0.1 μm, the carrier concentration of 1 × 10^17^, 1 × 10^16^, and 1 × 10^19^ cm^−3^, and bulk defect densities of 1 × 10^16^, 1 × 10^11^ and 1 × 10^16^ cm^−3^ had been found for CdS, CMTS, and SnS layer consecutively. The marked improvement in *V*_OC_ of 1.073 V by inserting the SnS BSF layer can be explained by decreasing the reverse saturation current and lowering the contact resistance with a smaller Schottky barrier height formed at *p*^*+*^-SnS/Pt interface.

The solar cell PCE of 25.43% was obtained for a single *n*-CdS/p-CMTS junction pristine solar cell (without BSF layer), which retained within the Shockley-Queisser limit (maximum efficiency of 30% at 1.1 eV [46], and 33.16% at 1.34 eV [47]). In 1961, Shockley-Queisser developed a theoretical limit of a single junction solar cell efficiency having a specific bandgap *E*_g_-based on the “principle of detailed balance theory” equating the incoming and outgoing photon fluxes with the light management system. However, in this study, the *PCE* of 31.51% was achieved by inserting an additional semiconductor of SnS as a BSF layer as *n*-CdS/*p*-CMTS/*p*^*+*^-SnS forming of *n-p-p+* double heterojunction cell. As observed in previous studies, this double junction cell efficiency improvement can be demonstrated by Tail-States-Assisted (TSA) two-steps photon upconversion process [[48], [49], [50]]. In the TSA process, two low-energy, i.e., sub-bandgap photons, are absorbed noticeably in a sequence by Urbach tail-states of materials that generate additional EHPs. Therefore, a semiconductor material (i.e., SnS as BSF) having a favorable band gap and doping concentration with a strong absorption coefficient could result in an efficient TSA upconversion process in a longer wavelength region [[48], [49], [50], [51]]. This is the primary origin of the high-efficiency *η* of over 31.51% observed in ZnO:Al/i-ZnO/CdS/Cu_2_MnSnS_4_ double heterojunction solar cells. Interestingly, the efficiency η of ∼42% under AM 1.5G solar illumination for single junction solar cells has recently been reported for nanostructured solar cells [52]. A comparative study of reported literature and proposed heterostructure are demonstrated in Table 2.

**Table 2:** A comparative study of proposed cells and comparison with the previously reported cells.

Solar cell structure	V_OC_ (V)	J_SC_ (mA/cm^2^)	FF (%)	ⴄ (%)	Ref.
Cu/ZnO:Al/i-ZnO/*n*-CdS/*p*-CMTS/Pt	0.883	34.41	83.74	25.43	Proposed (Pristine cell)
Cu/ZnO:Al/i-ZnO/*n*-CdS/*p*-CMTS/*p*^*+*^*-*SnS/Pt	1.074	36.21	81.04	31.51	Proposed (Cell with BSF)
i-ZnO/*n*-CdS/*p*-CMTS	0.88	24.10	77.90	16.50	[6]
AZO/n-ZnO/*n*-CdS/*p*-CBTS	0.78	11.64	74.77	6.9	[53]
AZO/i-ZnO/CdS/CMTS/Back contact	1.11	26.26	61.08	17.81	[20]
AZO/i-ZnO/Zn (O, S)/CMTS/Back contact	1.11	26.27	66.22	19.46	[20]
AZO/i-ZnO/SnS_2_/CMTS/Back contact	1.12	26.44	68.33	20.26	[20]

Herein, the CMTS absorber and SnS BSF have been demonstrated and utilized, which are inexpensive, economical, earth-abundant, and environmentally benign. However, these extensive simulation results revealed that the Cu_2_MnSn_4_ and SnS have strong potential as competitive photovoltaic materials with favorable band gaps for fabricating high-efficiency cost-competitive solar cells.

## 4 Conclusion

The impact of different parameters of CMTS TFSCs with CdS ETL and SnS BSF layers has been investigated systematically by the SCAPS-1D computer program. The impact of major influential PV parameters of layer thickness, carrier concertation, bulk, and interface defect density of photoactive materials, including operating temperature and metal contract effect, has been explored. The optimized parameters thickness of 0.05, 0.8, and 0.1 μm, the carrier concentration of 1 × 10^17^, 1 × 10^16^, and 1 × 10^19^ cm^−3^, and bulk defect density of 1 × 10^16^, 1 × 10^11^ and 1 × 10^16^ cm^−3^ for CdS, CMTS, and SnS layer consecutively. The *PCE* of 25.43% with the photocurrent *J*_SC_ of 34.412nullmA/cm^2^, open circuit voltage, *Voc* 0.882 V, and the fill factor, *FF* 83.74% were obtained from pristine CMTS solar cell without BSF of SnS, while the *PCE* of 31.51% and *J*_SC_, *V*_OC_, *FF* of 36.21nullmA/cm^2^, 1.073, and 81.04%, respectively with an inclusion of *p*^*+*^-SnS BSF layer. These comprehensive simulation results pave a solid direction for designing high performance CMTS photovoltaics. Furthermore, this study reveals Cu_2_MnSn_4_ and SnS semiconductors have strong potential as competitive photovoltaic materials for fabricating highly efficient cost-sensitive thin film solar cells.

## Author contribution statement

Ahmmad Isha, Abu Kowsar: Conceived and designed the experiments; Analysed and interpreted the data; wrote the paper. Abdul Kuddus: Analysed and interpreted the data; wrote the paper. M. Khalid Hossain, Md. Hasan Ali, Md. Dulal Haque: Contributed reagents, materials, analysis tools or data. Md. Ferdous Rahman: Performed the experiments; analysed, and interpreted the data.

## Data availability statement

Data will be made available on request.

## Declaration of interest's statement

The authors declare that they have no known competing financial interests or personal relationships that could have appeared to influence the work reported in this paper.

## Funding

The work is financially supported by the Bangladesh Council of Scientific and Industrial Research (BCSIR) regular R&D Scope (Ref. no: 39.02.0000.011.14.111.2019/228; Serial No. 41; Date: 06.11.2019).
